# Topological Measurements of DWI Tractography for Alzheimer's Disease Detection

**DOI:** 10.1155/2017/5271627

**Published:** 2017-03-02

**Authors:** Nicola Amoroso, Alfonso Monaco, Sabina Tangaro, Alzheimer's Disease Neuroimaging Initiative

**Affiliations:** ^1^Università degli Studi di Bari “A. Moro”, Via Orabona 4, 70123 Bari, Italy; ^2^Istituto Nazionale di Fisica Nucleare, Sezione di Bari, Via Orabona 4, 70123 Bari, Italy

## Abstract

Neurodegenerative diseases affect brain morphology and connectivity, making complex networks a suitable tool to investigate and model their effects. Because of its stereotyped pattern Alzheimer's disease (AD) is a natural benchmark for the study of novel methodologies. Several studies have investigated the network centrality and segregation changes induced by AD, especially with a single subject approach. In this work, a holistic perspective based on the application of multiplex network concepts is introduced. We define and assess a diagnostic score to characterize the brain topology and measure the disease effects on a mixed cohort of 52 normal controls (NC) and 47 AD patients, from Alzheimer's Disease Neuroimaging Initiative (ADNI). The proposed topological score allows an accurate NC-AD classification: the average area under the curve (AUC) is 95% and the 95% confidence interval is 92%–99%. Besides, the combination of topological information and structural measures, such as the hippocampal volumes, was also investigated. Topology is able to capture the disease signature of AD and, as the methodology is general, it can find interesting applications to enhance our insight into disease with more heterogeneous patterns.

## 1. Introduction

Recent years have shown an increasing interest for graph-based measures in magnetic resonance imaging (MRI) and diffusion-weighted imaging (DWI) studies focused on brain diseases [1–6]. Among neurodegenerative diseases, Alzheimer's disease (AD) is the most common type of dementia affecting over 5 million people [7, 8] and is characterized by a well-known stereotyped pattern involving a whole brain left privileged atrophy, especially affecting some regions related to cognitive functionality as the hippocampus [9–13]. However, it is not clear yet whether the combined use of MRI and DWI modalities can significantly enhance its diagnosis.

Previous machine learning studies, investigating mixed cohorts of normal controls (NC) and AD patients, have reported conflicting results, an even more evident effect with the inclusion of mild cognitive impairment (MCI) subjects. In some cases the combination of DWI and MRI features reported a significant classification improvement [14, 15]; in others these results were not confirmed [16]. It is obvious that a fair comparison should require common data sets and validation techniques; nevertheless, it is manifest that a primary role is played by the different features adopted. Different features, in fact, not only provide a different base of knowledge (which naturally affects the machine learning models) but also capture different clinical aspects. Measures based on directional diffusion, such as fractional anisotropy (FA), have been extensively used as they are able to detect the connectivity impairment effect of AD [17]. Some studies revealed remarkable effects with axial and radial diffusivity (*λ*_1_, RD) [18, 19]. In other cases huge effects were revealed in RD or mean diffusivity (MD) [20]. Finally, even if it is FA to be largely adopted, in some cases it can result in being insensitive [21, 22].

It is worth noting that the vast majority of reported results are focused on voxelwise DWI-related measures more than global connectivity metrics. However, the recent developments of more accurate and sophisticated processing pipelines for tractography reconstruction [23, 24] have encouraged the exploration of connectivity and topological measures to quantify the brain changes [25, 26]. Typical findings especially inherent to AD are related to connectivity disruption, eventually characterized by a loss of small world [27, 28] or rich club organization of the brain [29, 30]. AD patients exhibit a decreased network efficiency, implying abnormal topological organization [31, 32].

These studies are based on two, not necessarily competing, underlying hypotheses; that is, brain dysfunctions can be yielded by (i) a local connectivity impairment [33] or by (ii) an abnormal overall organization of the brain [34, 35]. The local impairment hypothesis has been largely confirmed. However, for the second hypothesis encouraging results have been reported. Indeed, topological measures can have detectable effect size [36, 37].

A holistic approach which describes the AD effects from a topological perspective is adopted here. More than focusing on local impairments we look for discriminating patterns in the brain connectivity organization; thus, DWI tractography is used to introduce a diagnostic topological score. As for the chosen cohort T1 MRI scans were also available; the score is compared and combined with volumetric measures to assess its informative content. The presented methodology is general, even tested in this case on Alzheimer's disease. It allows a description of the overall brain topology; thus, its application to diseases with less stereotyped patterns [38], such as Schizophrenia or Multiple Sclerosis, could give further insight.

## 2. Materials and Methods

### 2.1. Brain Connectivity Matrices

Data used in the preparation of this article were obtained from Alzheimer's Disease Neuroimaging Initiative (ADNI) database (http://adni.loni.usc.edu). The ADNI was launched in 2003 as a public-private partnership, led by Principal Investigator Michael W. Weiner, MD. The primary goal of ADNI has been to test whether serial magnetic resonance imaging (MRI), positron emission tomography (PET), other biological markers, and clinical and neuropsychological assessment can be combined to measure the progression of mild cognitive impairment (MCI) and early Alzheimer's disease (AD).

For the present study 99 subjects from Alzheimer's Disease Neuroimaging Initiative (ADNI) including normal controls (NC) and Alzheimer disease (AD) patients were analyzed. We chose this cohort in order to have for each subject both T1 MRI and DWI brain scans. T1-weighted sequences (voxel size = 1.2 × 1.0 × 1.0 mm^3^; TI = 400 ms; TR = 6.98 ms; TE = 2.85 ms; flip angle = 11) and DWI scans (voxel size = 2.7 × 2.7 × 2.7 mm^3^) are described in detail on the ADNI website (http://adni.loni.usc.edu/wp-content/uploads/2010/05/ADNI2_GE_3T_22.0_T2.pdf); in particular for DWI 46 separate images were acquired: 5 T2-weighted (*b* = 0 s/mm^2^ images) and 41 diffusion-weighted images (*b* = 1000 s/mm^2^). Demographics and clinical information are shown in Table 1.

**Figure 1:**
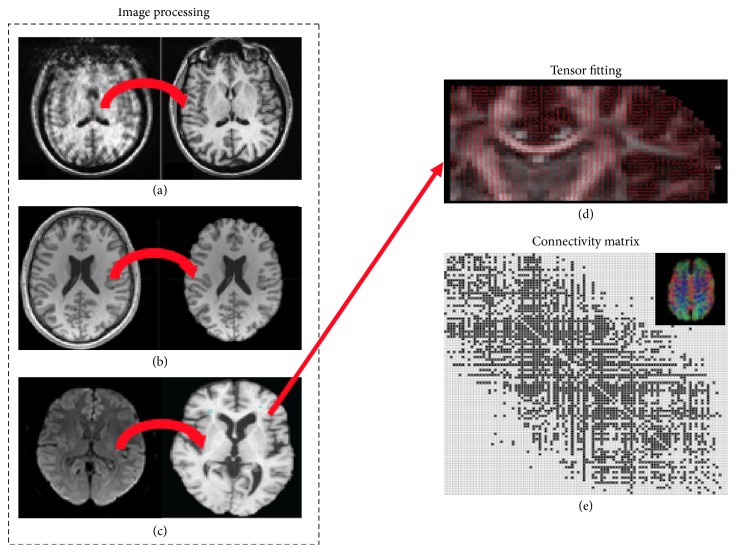
The figure shows the processing pipeline underwent by brain DWI scans. The dotted box includes the dedicated image processing steps: (a) eddy correction, (b) brain extraction, and (c) affine registration. For each voxel the diffusion tensor was estimated, (d) thus allowing the probabilistic fiber reconstruction. Using the Harvard-Oxford atlas, the connectivity matrix derived from tractography was computed for each subject.

For each subject DICOM images were acquired from ADNI database. MRICRON software was used to convert DICOM to NIFTI format, with the* dcm2nii* suite. Then FMRIB Software Library (FSL) by the Oxford Centre for Functional MRI of the Brain, and in particular its diffusion toolkit FDT, was used for the complete image processing pipeline; see Figure 1 for the overall flowchart:

1. Eddy current correction was performed to mitigate artifacts caused by eddy currents in the gradient coils.
2. Brain extraction was performed to erase nonbrain tissue from each subject scan, thus reducing the computational burden of the analysis.
3. An affine registration of all scans was employed to spatially normalize the whole data set to the MNI152 template. With this step the image processing phase was concluded.
4. Bayesian estimation of diffusion parameters and the inherent tensor fitting was obtained with sampling techniques at each voxel. This step was preparatory for running the subsequent probabilistic tractography.
5. Finally, probabilistic tractography was performed to obtain the connectivity matrix of each subject. Specifically, the Harvard-Oxford cortical atlas (http://neuro.imm.dtu.dk/wiki/Harvard-Oxford_Atlas) was used, thus resulting in a brain parcellation of 96 regions, 48 per hemisphere.

The final output was a weighted symmetric connectivity matrix *𝒲* whose elements *w*_*ij*_ represented the strength of connectivity, that is, the number of fibers, between the *i*th and *j*th regions. The fundamental step of the whole image processing was the fiber reconstruction. The FDT tool generates a probabilistic streamline or a sample from the distribution on the location of the true streamline. By taking many such samples the histogram of the posterior distribution on the streamline location or the connectivity distribution is then built. Finally, the most probable traits connecting two regions are computed. We averaged the traits connecting region *i* to *j* and vice versa *j* to *i* to obtain a symmetric matrix. We considered all non-null connections, disregarding the weight information and obtaining a binary connectivity matrix *𝒞* whose elements *c*_*ij*_ were straightforwardly defined:

(1) $$\begin{array}{c} c_{ij}=\left\{\begin{array}{cc} 1 & if\;\;w_{ij}>0\\ 0 & otherwise. \end{array}\right. \end{array}$$

As the focus of this study was the topological organization of the brain, we privileged the study of *𝒞*; nonetheless, we also investigated the information carried by the connectivity weights. In principle, weight information should help the cohort discrimination as weights are directly affected by the impairment caused by the disease. However, it is worthwhile to note that tractography is very sensitive to artifacts and noise due to reconstruction algorithms and as a consequence it sometimes shows biological insights difficult to interpret.

### 2.2. Topological Overlap

The binary connectivity matrix *𝒞*^*α*^ of each subject *α* in the cohort is a compact representation of connected brain regions. A reasonable and partially confirmed hypothesis, deriving from the AD peculiarity of being a neurodegenerative disease, is that connectivity impairment should have a direct effect on the network topology. Besides, the impairment should reflect the severity of the pathological condition; thus, it should be expected that, for severe AD conditions, topology should manifest more evident changes. Nonetheless, natural biological variability can sometimes conceal these local effects and a huge statistical power will be required to investigate each brain connection and get a significant measurement.

We propose instead of describing the connectivity loss with a global indicator, trying to capture the whole brain behavior. To capture the whole informative content of the cohort in one comprehensive model we chose to adopt the novel multiplex network framework. A multiplex network, from now onward simply multiplex, is by definition a collection of networks sharing the same nodes [39]. Generally adopted in social sciences, this concept is naturally introduced to describe system with heterogeneous interactions. As an example, scientific authors with a common publication can be represented as a network; if this operation is stratified considering, for example, different journals or editors, a multiplex description arises. Another common example concerns the different relationships a group of people can share: social, geographical, and physical, just to mention a few.

**Figure 2:**
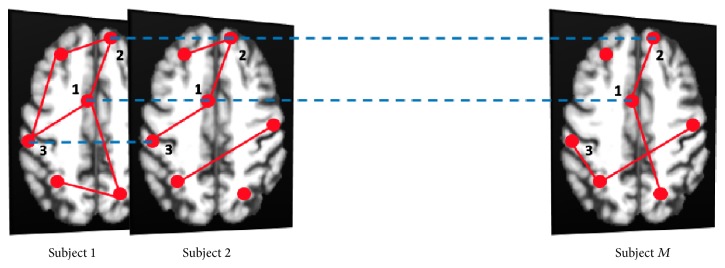
All subjects of the cohort are represented through graphs with exactly the same nodes (in red), corresponding to brain anatomical districts, but with different connections. For example, the figure shows the case of two nodes (1, 2) connected for all subjects and two nodes (1, 3) connected only in the first two subjects. The presence of a link in different subjects is also outlined (blue dashed lines). For each link in the networks it is possible to measure the fraction of subjects having a link in common, the so-called link overlap.

The same concept applies here if we consider the anatomical districts as the fixed nodes of a network and build a network for each subject as if subjects were representing a stratification factor. Given a collection of these single subject networks, the multiplex can be visually represented as a 3D structure formed by *M* layers, one layer for each subject *α*, as shown in Figure 2.

Let *C* = (*𝒞*^1^,…, *𝒞*^*α*^,…, *𝒞*^*M*^) be the multiplex with each single subject graph *𝒞*^*α*^ formed by *N* nodes, the 96 labeled regions of the Harvard-Oxford atlas, and *M* layers (layers and subjects in this work will be interchangeably used). For a generic node of the multiplex *i* and for two generic subjects *α* and *α*′ it is possible to define the local node overlap *n*_*i*_^*α*,*α*′^ [40] which is the total number of nodes *j* linked to the node *i* in a couple of layers *α* and *α*′:

(2) $$\begin{array}{c} n_{i}^{\alpha \alpha^{\prime }}=\overset{N}{\underset{j=1}{\sum }}c_{ij}^{\alpha }c_{ij}^{\alpha^{\prime }}. \end{array}$$

This is really useful information when investigating how central the node is within each layer, for example, to understand if there is a direct association between the kind of relationship defining the layer and the role played within it by a particular node.

However, from a topological point of view this is not very useful information, because what defines topology is not how intense the connections are, but their existence. Thus, adopting the same strategy to our case, we introduce here the link overlap matrix *𝒪* and its elements *o*_*ij*_:

(3) $$\begin{array}{c} o_{ij}=\frac{1}{M}\overset{M}{\underset{\alpha =1}{\sum }}c_{ij}^{\alpha }. \end{array}$$

This matrix counts the number of times a link is present within each layer *M*. It is therefore a symmetric matrix whose values lie in the [0,1] interval.

It is reasonable to expect that link overlap should characterize important correlations among the different layers. One of the questions addressed by the present work is whether this measurement can detect the cross-sectional differences within a mixed NC/AD cohort. Accordingly, we built the multiplexes of NC subjects and AD patients. For both cases, the link overlap matrices *𝒪*_*𝒩𝒞*_ and *𝒪*_*𝒜𝒟*_ were computed. These matrices became binary with a 0.5 threshold for both NC and AD cohorts, considering it a likelihood measure assigned to each link.

**Figure 3:**
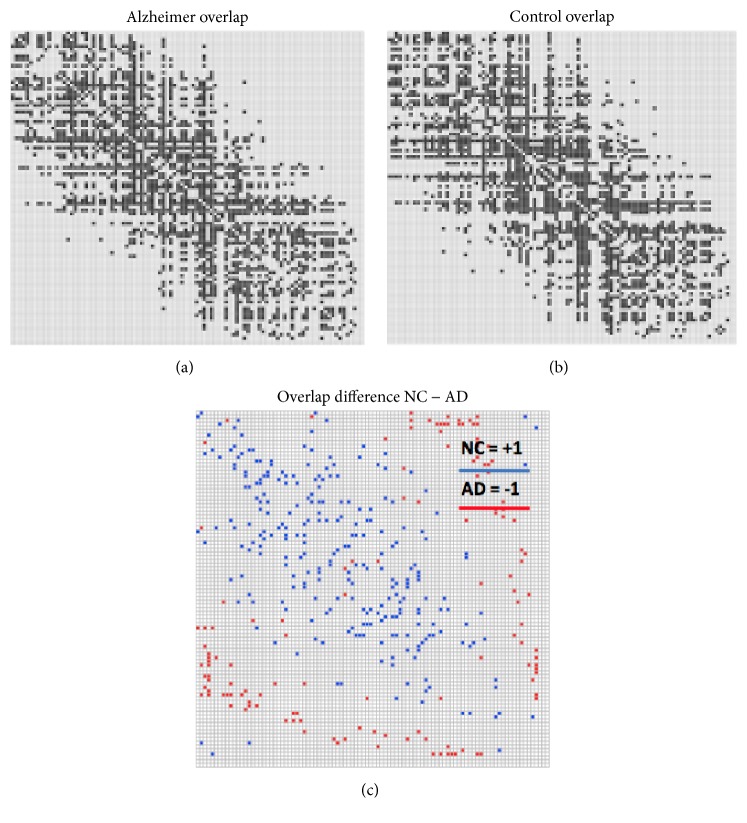
The figure shows the overlap matrices computed for the AD (a) and the NC (b) cohorts represented by the angular order of the eigenvectors. (c) shows the overlap difference between the controls and patients. AD patients have a lesser number of edges *E*_AD_ = 1463 < 1523 = *E*_NC_. Interestingly, AD and NC seem to show different patterns of connectivity more than an overall impairment.

The link overlap matrices represent the connectivity backbone of each population; in fact a qualitative difference can be directly observed by comparing the NC and the AD cases as shown Figure 3.

The overlap difference matrix *𝒟* defined as

(4) $$\begin{array}{c} D=O_{NC}-O_{AD} \end{array}$$

has some interesting properties. It is a symmetric matrix whose elements *d*_*i*,*j*_ are 0 for all connections with an identical behavior in both NC and AD cohorts and ±1 for those connections present, respectively, only in *𝒪*_*𝒩𝒞*_ or *𝒪*_*𝒜𝒟*_. To emphasize these differences we introduce for each subject *α* a topological connectivity score *𝒮*^*α*^ as the Hadamard, that is, element-wise, product of *𝒞*^*α*^ and *𝒟*:

(5) $$\begin{array}{c} S^{\alpha }=\overset{N}{\underset{i,j=1}{\sum }}\frac{1}{2}c_{ij}^{\alpha }d_{ij} \end{array}$$

with *d*_*ij*_ representing the elements of *𝒟* and the division by 2 takes into account the symmetry of *𝒞*^*α*^ and *𝒟*. In the same way a weighted connectivity score *𝒮*_*w*_^*α*^ can be introduced by considering in the previous equation the original connectivity matrix *𝒲*^*α*^ and its elements *w*_*ij*_:

(6) $$\begin{array}{c} S_{w}^{\alpha }=\overset{N}{\underset{i,j=1}{\sum }}\frac{1}{2}w_{ij}^{\alpha }d_{ij}. \end{array}$$

The topological score is designed to capture how disease affects the topological organization of the brain. Its weighted version, which includes the information inherent to the connectivity strength, could in principle enhance the segregation capability of the two cohorts. In fact, we will directly address this aspect in the following sections. The two scores were finally normalized to get a direct probabilistic interpretation as diagnostic scores.

## 3. Results and Discussion

### 3.1. Quantitative Assessment of *𝒮* and *𝒮*_*w*_ Scores

**Figure 4:**
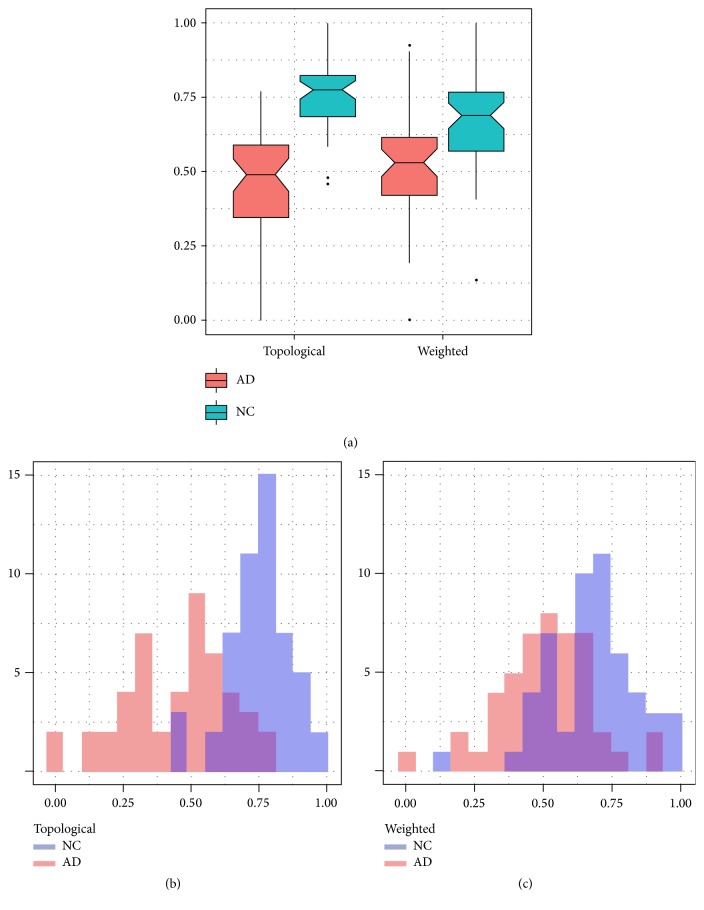
The figure shows (a) the boxplot of topological *𝒮* and weighted *𝒮*_*w*_ scores. The separation effect is more evident when using *𝒮*. This is also evident when looking at the score distributions: the weighted score (c) shows a consistent overlap between NC and AD if compared with topological score distribution (b).

To evaluate the capability of both *𝒮* and *𝒮*_*w*_ to capture the effects yielded by disease on brain organization we computed them adopting a leave-one-out cross-validation framework. Thus, each score was computed using the difference overlap *𝒟* resulting from the remaining subjects in the cohort. The separation between AD and NC, as shown in Figure 4, denoted a significant effect.

In fact, the topological score *𝒮* resulted in a Wilcoxon *p* value *p* = 2 · 10^−13^ while for *𝒮*_*w*_ we found *p*_*w*_ = 2 · 10^−11^. Even if both *p* values showed a 0.01 significance, the relative effect measured in terms of Cohen's *h* distance revealed that *𝒮* had a larger effect, almost double, than *𝒮*_*w*_ with *h* = 1.4 > 0.8 = *h*_*w*_. The effect was also qualitatively manifest when comparing the score distributions, shown in Figures 4(b) and 4(c). The weighted scores of NC and AD showed a greater superimposition if compared with topological scores.

**Figure 5:**
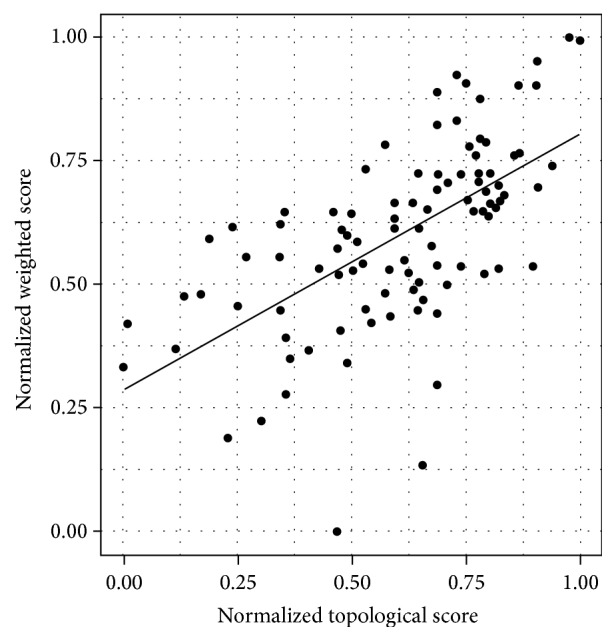
The figure shows moderate Pearson's *r* = 0.61 correlation characterizing the topological score *𝒮* and its weighted variant *𝒮*_*w*_. A higher correlation could have been expected; nonetheless, artifacts and noise yielded by reconstruction tractography algorithms have obviously a greater effect on computed weights, more than their presence.

These results demonstrated that the proposed topological scores had a significant association with the disease effect, or in other words, they were proper measurement of the topological differentiation affecting a diseased brain. Provided that the topological score resulted in a diagnostic index being more effective than its weighted variant, they were obviously correlated measures, as shown in Figure 5.

However, their Pearson's correlation *r* = 0.61 was not so high as one could have expected. This result showed that the information carried by both the scores was not redundant. Besides, this result can be interpreted in terms of the quality of the information content carried by both scores. Interestingly, the topological score furnishes better quality information even disregarding the additional weight information. Nonetheless, the weighted topological score should deserve further studies, especially aimed at removing, as previously explained, noisy connections and artifacts yielded by the tractography reconstruction algorithms which obviously negatively affected its discriminating power.

### 3.2. Brain Topology and Anatomy

Another important aspect concerning the topological score interpretation and its weighted version is whether they can or cannot directly be related to brain anatomy. This analysis in particular aims at quantifying whether an association exists from the topological organization of the brain and the atrophy of particular brain regions related to the disease. This test should outline in particular how structural MRI and DWI can be combined to better characterize and distinguish the diseased patterns.

**Figure 6:**
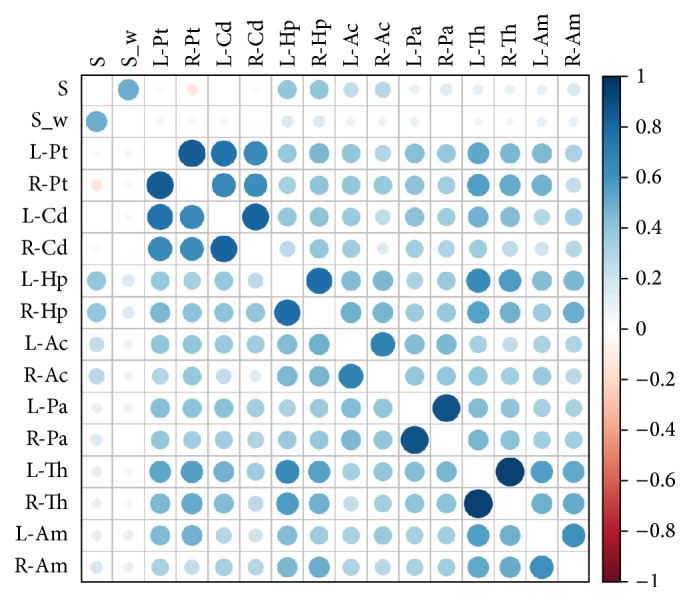
The figure shows Pearson's correlation between the proposed topological *𝒮* and weighted *𝒮*_*w*_ scores and the structural measurements of Left Thalamus (L-Th), Left-Caudate (L-Cd), Left Putamen (L-Pt), Left Pallidum (L-Pa), Left Hippocampus (L-Hp), Left Amygdala (L-Am), Left Accumbens (L-Ac), Right Thalamus (R-Th), Right Caudate (R-Cd), Right Putamen (R-Pt), Right Pallidum (R-Pa), Right Hippocampus (R-Hp), Right Amygdala (R-Am), and Right Accumbens (R-Ac).

Firstly, we computed the volumes of subcortical features of interest for AD. Specifically, we measured the volumes of Left Thalamus (L-Th), Left-Caudate (L-Cd), Left Putamen (L-Pt), Left Pallidum (L-Pa), Left Hippocampus (L-Hp), Left Amygdala (L-Am), Left Accumbens (L-Ac), Right Thalamus (R-Th), Right Caudate (R-Cd), Right Putamen (R-Pt), Right Pallidum (R-Pa), Right Hippocampus (R-Hp), Right Amygdala (R-Am), and Right Accumbens (R-Ac) with the FSL* FAST* tool. Then we measured Pearson's correlations between each regions and our proposed scores *𝒮* and *𝒮*_*w*_. Results are shown in Figure 6.

The correlations were ordered by hierarchical clustering, in this way the more correlated regions tended to be placed together in the correlation matrix. This is the reason, for example, for the manifest pairing of left/right regions. It is worth noting that both *𝒮* and *𝒮*_*w*_ were poorly correlated to structural features. This result would suggest that the topological brain organization contains intrinsic information that it does not share with structural measurements. The most correlated structural features to the proposed scores (*r* ~ 0.3) were the hippocampal volumes.

**Figure 7:**
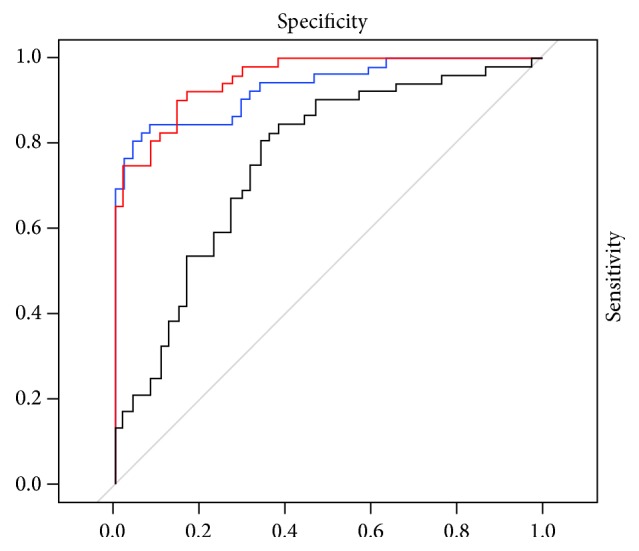
A comparison of the receiver operating characteristic curves for *𝒮* and *𝒮*_*w*_ scores (red), the structural features (black), and their combination (blue) is presented. Corresponding AUC performance is 95%, 93%, and 76%.

To measure the information content provided by *𝒮* and *𝒮*_*w*_ we trained with both of them (as we previously demonstrated they were not redundant) a support vector machine model with 500 5-fold cross-validation. Obviously, to avoid any bias in this step the computation of matrices *𝒪*_NC_, *𝒪*_AD_, and *𝒟* was performed again, but considering only the training sample. This test allowed also assessing the information contained in *𝒮* and *𝒮*_*w*_, when compared with the structural features derived from T1 scans. For this measure we adopted the area under the curve (AUC) of the receiver operating characteristic (ROC) curve. Results are summarized in Figure 7.

The average AUC corresponding to *𝒮* and *𝒮*_*w*_ scores was 95% with a 95% confidence interval of 92%–99%. For what concerns structural features the performance had a drastic drop with an AUC of 76% and confidence interval of 66%–86%. Interestingly, when combining the information of structural features with the topological one not a significant effect shows up. In fact, AUC was 93% with a 95% confidence interval of 0.88–0.98.

Structural and topological features are not correlated as shown in Figure 6; therefore, one could expect an improvement of classification when combining the two typologies of features. However, as previously mentioned, this is still an open question. For what concerns this study, these results made us hypothesize that there could be a misleading effect driven by confounding features. To test this hypothesis we used among structural features only hippocampal volumes; the measured AUC (88%, 95% confidence interval 75%–91%) was slightly higher than using the whole set of structural features; its effect also improved, even if not significantly, the overall classification performance with topological features (AUC 97%, 95% confidence interval 94%–100%).

This result suggests that a careful feature selection strategy should be applied to gain an effective information contribution from different imaging modalities.

## 4. Conclusions

In this study a novel approach to characterize the brain organization from a topological perspective is presented. In particular, because of the well-known and stereotyped pattern characterizing AD, we chose to use this pathology as a benchmark. A topological score and a weighted variant have been defined and used to train support vector machines on a mixed NC/AD cohort. Results showed that topological information was able to efficiently detect diseased patterns (AUC = 95%, 95% confidence interval 92%–99%).

We also addressed in this study the problem of quantifying the effect of combining MRI-based features with topological ones. We found that their combination can improve classification accuracy; nonetheless, this is strictly related to the quality of structural features used. In fact, when using all MRI features available the classification performance decreased; on the contrary, it was slightly raised using hippocampal volumes whose association with AD is well known. A subtle effect should be better investigated on larger cohorts.

The performance obtained is comparable with best results reported in the literature so far, but possible improvements could include a more refined study of weighted networks, instead of their binary version; nevertheless, this cannot be considered a limitation of the present study, whose main goal was to investigate the brain topology and understand whether the topological measures proposed were suitable for clinical purposes.

The presented methodology is general, even if in this case it has been tailored on Alzheimer's disease. For future work, we propose to investigate the application of this methodology to mixed cohorts including also MCI subjects, trying to tackle the discrimination problem between subjects converting to AD or not, and the early diagnosis of AD. Patients affected by neurodegenerative diseases incur a cognitive impairment which could be effectively diagnosed and monitored by these measurements, a useful trait for technological innovations in the e-health field, for example, for remote medicine applications, or for pharmacological industries, aiming at the development of drug therapies and clinical trials. Further investigations could be aimed at diseases affecting the brain organization with less stereotyped patterns.

## Figures and Tables

**Table 1:** Data size, age range, gender, and a cognitive score (Mini Mental State Examination (MMSE)) are shown for each diagnostic group: normal control (NC) and Alzheimer's disease (AD) subjects. Mean and standard deviation are shown when appropriated.

	Size	Age	Gender	MMSE
NC	52	73 ± 6	M/F 26/28	29 ± 1
AD	47	75 ± 9	M/F 29/18	23 ± 2
